# Suits for Mal-practice

**Published:** 1871-04

**Authors:** 


### Suits for Mal-practice.

We observe that Prof. S. D. Gross, and Dr. Gross, Jr., were before Judge Linn for mal-practice on the person of a young fisher, who died as the result of an operation for aneurism. After hearing the complaint, the Court entered a *non-suit* in the complaint. Pity the complainants had not aneurism operated upon with similar results.

